# The complete mitochondrial genome of the edible mushroom *Grifola frondosa*

**DOI:** 10.1080/23802359.2021.1917312

**Published:** 2022-01-27

**Authors:** Ying Song, Jianing Wan, Jun-Jun Shang, Zhan Feng, Yuchang Jin, Hewen Li, Ting Guo, Ying-Ying Wu, Da-Peng Bao, Min Zhang, Litao Lv, Junjie Liu, Rui-Heng Yang

**Affiliations:** a Institute of Edible Fungi, Liaoning Academy of Agricultural Sciences, Shenyang, PR China; b National Engineering Research Center of Edible Fungi, Ministry of Science and Technology (MOST), Key Laboratory of Edible Fungi Resources and Utilization (South), Ministry of Agriculture, Institute of Edible Fungi, Shanghai Academy of Agricultural Sciences, Shanghai, PR China; c Jiangsu China Green Co. Ltd, Siyang, PR China

**Keywords:** Mitochondrial genome, *Grifola frondosa*, Illumina sequencing, phylogenetic analyses

## Abstract

The culinary-medicinal mushroom *Grifola frondosa* is widely cultivated in East Asia. In this study, the complete mitochondrial genome of *G. frondosa* was determined using Illumina sequencing. The circular molecule was 197,486 bp in length with a content of 25.01% GC, which was one of the largest mitochondrial genomes in the order Polyporales. A total of 39 known genes encoding 13 common mitochondrial genes, 24 tRNA genes, 1 ribosomal protein s3 gene (*rps3*), and 1 DNA polymerase gene (*dpo*) were predicted in this genome. The phylogenetic analysis showed that *G. frondosa* clustered together with *Sparassis crispa*, *Laetiporus sulphureus*, *Wolfiporia cocos*, and *Taiwanofungus camphoratus*. The complete mitochondrial genome reported here may provide new insight into genetic information and evolution for further studies.

*Grifola frondosa* (Dicks.) Gray, commonly known as ‘maitake,’ is a delicious and culinary-medicinal mushroom. It is rich in many bioactive compounds, such as polysaccharides, proteins, and nonvolatile taste components (Huang et al. 2011; Liu et al. 2020). Mitochondria and mitochondrial DNA play important roles in generation of the energy, aging, and other physiological processes (Westermann and Prokisch 2002). Mitochondrial DNA was widely used for phylogeny, evolution, and strain discrimination for fungi (Fischer and Seefelder 1995; Wang et al. 2019; Li et al. 2020), which may be a benefit to taxonomy, variety protection, and breeding of *G. frondosa*. The expressions of mitochondrial proteins were also associated with fruiting-body development of *G. frondosa* (Numata et al. 2019). However, there is no information from the mitochondrial genome of *G. fro*ndosa reported. The complete mitochondrial genome reported here may provide important genetic information for further studies.

The dikaryoitc strain JPH3 used in this study is a widely cultivated strain (collected from Shanghai). Fermentation cultivation of fungal mycelium, DNA extraction, and genome sequencing was conducted according to the methods published previously (Yang et al. 2016, 2017; Wu et al. 2018). Finally, a total of 6.64 Gb raw data generated from the platform Hiseq4000. After sequence processing, the filtered reads were assembled into the circle genome using the software GetOrganelle (Jin et al. 2020). And the gene prediction and annotation were performed using MFannot (http://megasun.bch.umontreal.ca/cgi-bin/mfannot/mfannotInterface.pl). The neighbor-joining phylogenetic analysis combined with 35 other species belonged to Basidiomycota was performed using MEGA version 7.0, Tokyo, Japan (Kumar et al. 2016) with a Poisson model and 1000 bootstrap replicates.

The circular mitochondrial genome was 197,486 bp in length with a GC content of 25.01%, which was one of the largest mitochondrial genome in the order Polyporales (<*Phanerochaete carnosa*, 206,437 bp, Wang et al. 2020). A total of 108 protein-coding genes, including 13 conserved protein genes, 69 hypothetical protein genes, 24 tRNA genes, 1 ribosomal protein 3 gene (*rps3*), and 1 DNA polymerase gene (*dpo*), were detected in the mitochondrial genome. The 13 conserved protein genes consisted of 6 nicotinamide adenine dinucleotide dehydrogenase genes (*nad 1-6*), 3 ATP synthase (*atp6, apt 8*, and *apt 9*), 3 cytochrome oxidase genes (*cox 1–3*), and 1 apocytochrome b (*cob*). A total of 35 Group I introns, 1 Group II and 5 unclassified invaded into 8 genes, e.g. *cob* (11 introns), *cox1* (9 introns), *cox2* (4 introns), *cox3* (3 introns), *nad1* (2 introns), *nad2* (3 introns), *nad4* (1 introns), and *nad5* (8 introns). Moreover, this mitogenome contained 24 tRNA genes coding for all 20 standard amino acids.

Based on the 13 conserved proteins, the neighbor-joining phylogenetic tree revealed that *G. frondosa* (Grifolaceae) distributed in the clade containing the species *Sparassis crispa*, *Laetiporus sulphureus*, *Wolfiporia cocos*, and *Taiwanofungus camphoratus* (Figure 1). The results were in agreement with the previous report (Justo et al. 2017). The mitochondrial genome of *G. frondosa* would contribute to the understanding of the phylogeny and evolution of Polyporales.

**Figure 1.**
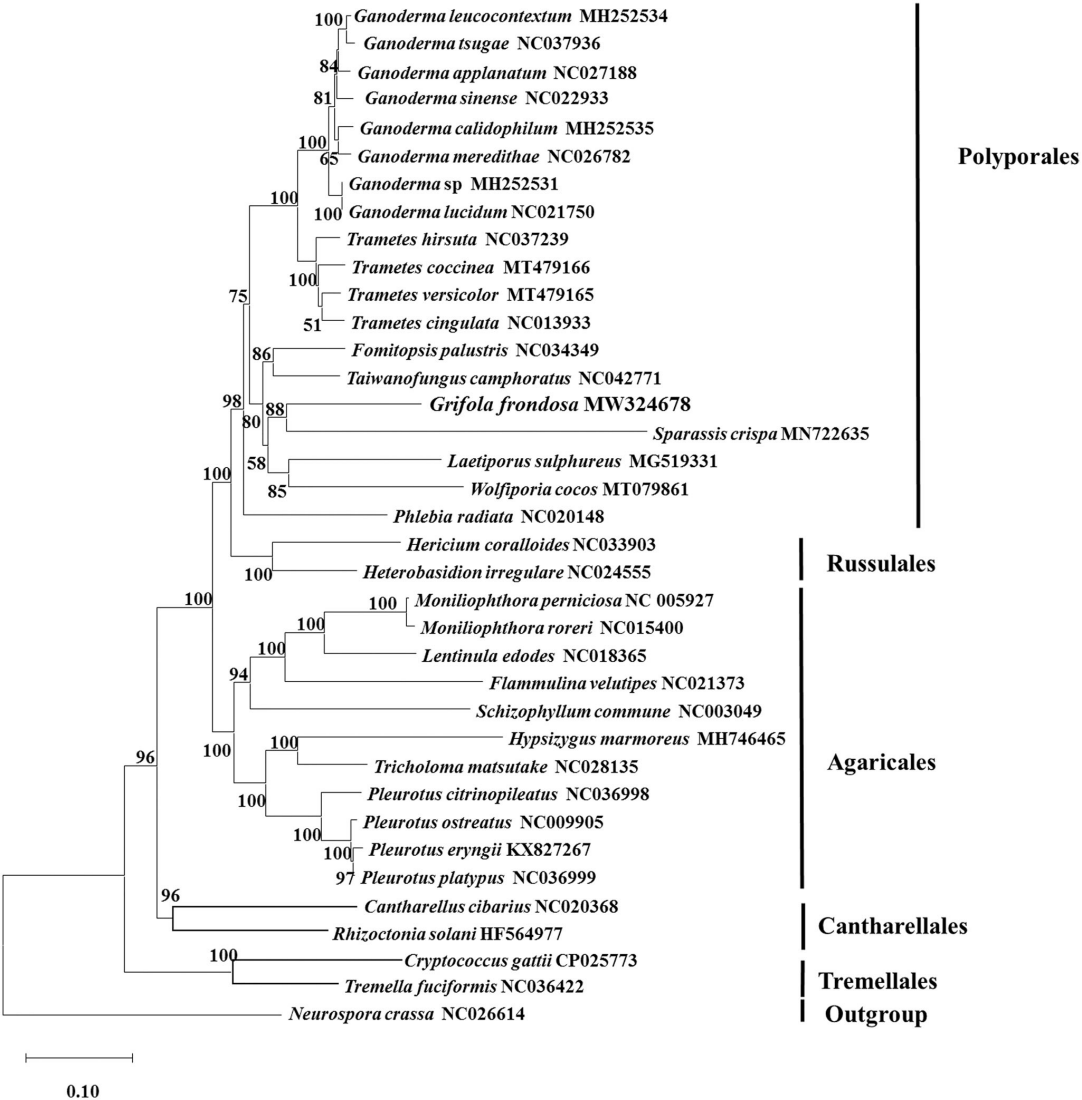
Phylogenetic analysis of 35 species of Basidiomycota conducted by neighbor-joining method based on concatenated amino acid sequences of 13 conserved protein-coding genes (common in all the species), including *atp6*, *atp8*, *atp9*, *cob*, *cox1*, *cox2*, *cox3*, *nad1*, *nad2*, *nad3*, *nad4*, *nad5*, and *nad6*. *Neurospora crassa* (NC_026614) was served as outgroup. The bootstrap support values were shown at each node.

## Data Availability

The genome sequence data that support the findings of this study are openly available in GenBank of NCBI at (https://www.ncbi.nlm.nih.gov/) under the accession number MW324678. The associated BioProject, Biosample, and SRA of numbers are PRJNA703746, SAMN18022987, and SRR13759100, respectively. The strain used in this study was deposited at Guangdong Microbial Culture Collection Center (GDMCC, http://www.gimcc.net/, gdmcc@gdim.cn) under the number GDMCC5.625.
